# Synovial sarcoma presenting with huge mediastinal mass: a case report and review of literature

**DOI:** 10.1186/1756-0500-6-240

**Published:** 2013-06-25

**Authors:** Samer Salah, Akram Al-Ibraheem, Amal Daboor, Maysa Al-Hussaini

**Affiliations:** 1 Department of medical oncology, King Hussein cancer center, Amman, Jordan; 2 Department of radiology, nuclear imaging division, King Hussein cancer center, Amman, Jordan; 3 Department of pathology and laboratory medicine, King Hussein cancer center, Amman, Jordan

**Keywords:** Mediastinal mass, Synovial sarcoma, Chemotherapy, Surgical resection

## Abstract

**Background:**

Synovial sarcoma presenting in the mediastinum is exceedingly rare. Furthermore, data addressing optimal therapy is limited. Herein we present a case where an attempt to downsize the tumor to a resectable state with chemotherapy was employed.

**Case presentation:**

A 32 year female presented with massive pericardial effusion and unresectable huge mediastinal mass. Computed axial tomography scan - guided biopsy with adjunctive immunostains and molecular studies confirmed a diagnosis of synovial sarcoma. Following three cycles of combination Ifosfamide and doxorubicin chemotherapy, no response was demonstrated. The patient refused further therapy and had progression of her disease 4 months following the last cycle.

**Conclusion:**

Synovial sarcoma presenting with unresectable mediastinal mass carry a poor prognosis. Up to the best of our knowledge there are only four previous reports where primary chemotherapy was employed, unfortunately; none of these cases had subsequent complete surgical resection. Identification of the best treatment strategy for patients with unresectable disease is warranted. Our case can be of benefit to medical oncologists and thoracic surgeons who might be faced with this unique and exceedingly rare clinical scenario.

## Background

Soft tissue sarcomas (STS) are rare malignant tumors that comprise less than 1% of malignant neoplasms. In the thorax, where the majority of malignant tumors are carcinomas, the proportion of STS is even much less; probably less than 0.01% of all malignant thoracic neoplasms [1]. A population based study demonstrated that 17% of new cases of STS including variety of histologies arise in the thorax, accounting for an approximate incidence of primary thoracic STS of 6 per million populations [2]. These tumors may arise in variety of locations including lung, mediastinum, heart, and pleura.

The mediastinum as a primary site of occurrence of synovial sarcoma is exceedingly rare. Although complete resection had been the only therapy associated with long term survival, uncertainty exists regarding the best therapeutic strategy for patients with unresectable disease. External beam radiotherapy (EBRT) is the most commonly employed primary therapy when the mass is unresectable [3,4]. Chemotherapy as a primary therapy, on the other hand, is only rarely reported [5]; there are only four previous reports where chemotherapy was employed as the primary therapy amongst 16 reported cases of unresectable primary mediastinal synovial sarcoma (Table 1). In this case report, we present the outcome following combination chemotherapy that was delivered as a primary therapy in an attempt to downsize the tumor to achieve resectability, and review the available literature in an attempt to shed light on the available therapeutic options for this kind of clinical presentation.

**Table 1:** Primary mediastinal synovial sarcoma patients presenting with unresectable/ advanced disease

**Author**	**Age/ Gender**	**Presentation**	**Histologic subtype**	**Treatment**	**Outcome**
de Zwaan C, et al. Neth Heart J. 2007;15(6):226–8.	19 / M	S.O.B, congested neck veins, edema, cardiac tamponade	M	Patient refused therapy	DWD at 3 months.
Korula A, et al. Singapore Med J. 2009 Jan;50(1):e26-8.	49 / M	S.O.B, chest pain, Pericardial effusion	NA	Supportive care only. Patient refused CTX	NA
Suster S, et al. Am J Surg Pathol. 2005 May;29(5):569–78.	13 / M	Weakness, S.O.B, right pleural effusion	M	EBRT only	DWD at 6 months after developing liver metastases.
	23 / F	Weakness, chest pain	B	Partial excision followed by EBRT	NA
	48 / M	Chest pain	M	EBRT only	NA
	54 / F	Left shoulder pain	B	Partial excision followed by EBRT	Local progression and epidural metastasis at one year. DWD.
	83 / M	S.O.B, pleural effusion	M	EBRT only	NA
Arafah M, et al. Indian J Pathol Microbiol. 2011 Apr-Jun;54(2):384–7.	30 / M	S.O.B, cough, weight loss	M	Palliative CTX (Ifosfamide+Doxorubicin)	Regression of mediastinal mass after 3 cycles. AWD at 6 months
Hung JJ, et al. Ann Thorac Surg. 2008 Jun;85(6):2120–2.	24 / M	Chest pain, S.O.B, back pain, cough, fevers	M	Partial excision followed by EBRT	PD five weeks after EBRT (local+lung metastases). PD with bone metastases following treatment with Ifosfamide and epirubicin. DWD 6 months after diagnosis.
Paquette M, et al. J Thorac Oncol. 2010 Jun;5(6):898–906.	NA	NA	NA	EBRT only	PR following EBRT. Local progression at 32 months. DWD at 38 months.
	NA	NA	NA	CTX (Ifosfamide+Doxorubicin)	PD during CTX. DWD at 5 months
Trupiano JK, et al. Ann Thorac Surg. 2002 Feb;73(2):628–30.	30 / F	Incidental on imaging	B	Partial resection and combination CTX	DWD at 10 months
Nakagawa Y, et al. Nihon Kokyuki Gakkai Zasshi. 2010 Oct;48(10):734–8.	65 / M	Cough with bloody sputum	NA	CTX, EBRT, and hyperthermia	PD following CTX, EBRT, and hyperthermia. DWD at 20 months.
Lee HJ, et al. J Lung Cancer 2008;7(1):29-33	44 / M	Cough, SOB, chest pain	M	Partial resection following PR to neoadjuvant Adriamycin+ Dacarbazine	Adjuvant EBRT (59.4 Gy in 33 fractions). Developed bilateral lung metastases three months after finishing EBRT.
Kaira K, et al. J Comput Assist Tomogr. 2008 Mar-Apr;32(2):238-41	64 / F	Back pain, dysphagia	NA	EBRT, CTX upon PD (Ifosfamide+Doxorubicin), re-irradiation and hyperthermia	Symptomatic improvement after EBRT, response to CTX, DWD at 24 months
	58 / M	Right sided back pain	NA	EBRT and hyperthermia, CTX (Ifosfamide+Doxorubicin) upon PD, second line CTX (Gemcitabine + Docetaxel), third line carboplatin	Initial response after EBRT, hyperthermia, and first line CTX, DWD at 19 months
Current case	32 / F	S.O.B, lower limb edema, pericardial effusion	M	CTX (Ifosfamide +Doxorubicin)	PD after CTX, refused further therapy. AWD at 11 months

*S*.*O*.*B* shortness of breath, *NA* not available, *M* monophasic, *B* biphasic, *CTX* chemotherapy, *EBRT* external beam radiotherapy, *DWD* died with disease, *AWD* alive with disease, *PD* progression of disease, *PR* partial response.

## Case presentation

**Figure 1:**
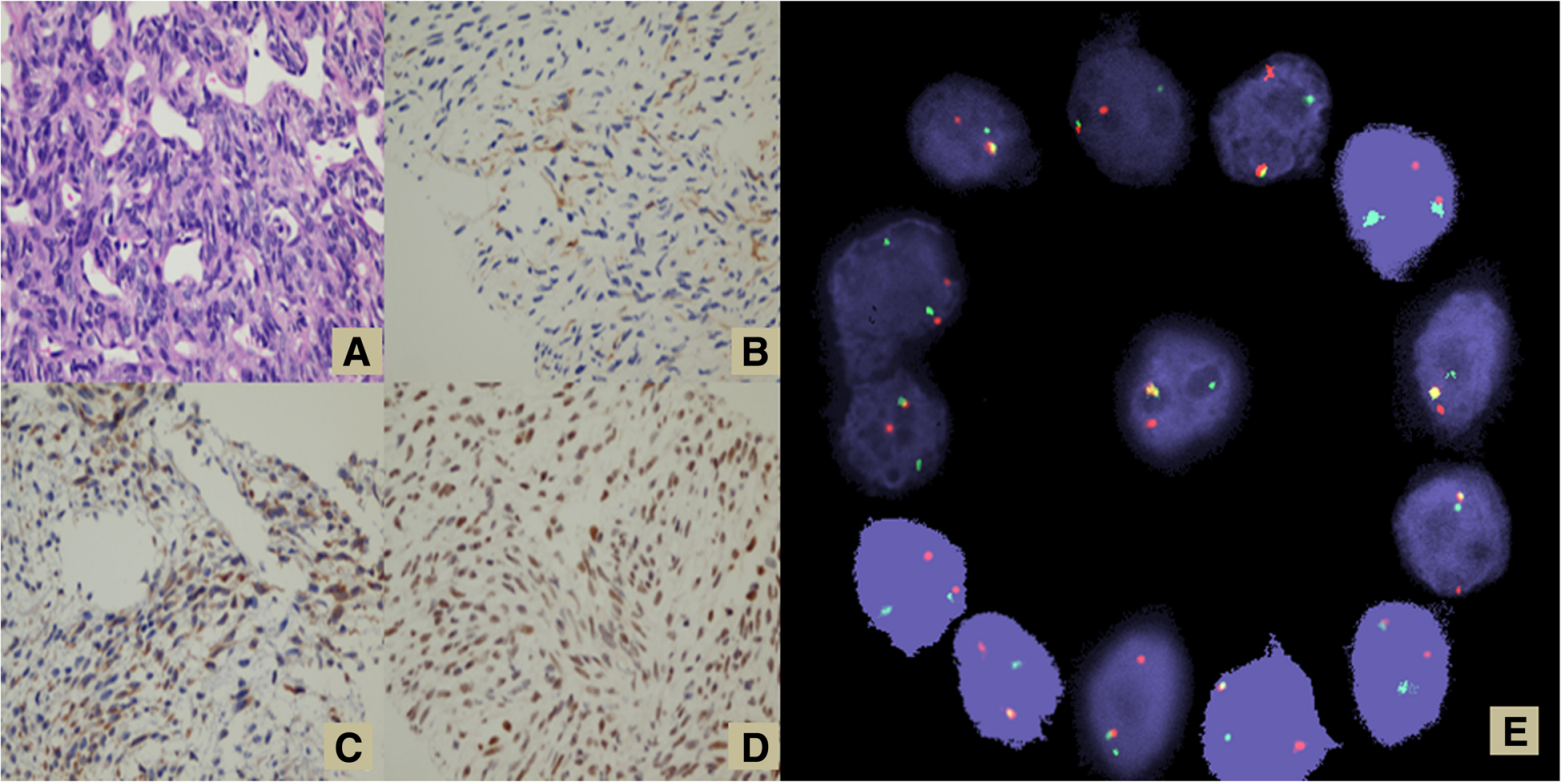
**Pathologic examination of the mediastinal mass biopsy.** (**A**) The tumor shows proliferation of oval cells separated by hemangiopericytomatous vessels (H&E X40). (**B**) Scattered positivity in tumor cells for pancytokeratin (MNF) can be seen (X20). (**C**) Cytoplasmic positivity for Bcl-2 is appreciated (X40). (**D**) Fli-1 nuclear stain is seen in this case (X40). CD99 was completely negative (not shown). (**E**) Abnormal cells hybridized with LSI SS18 (18q11.2) Dual Color, Break Apart Rearrangement Probe. The cells in this image show one fusion, one orange, and one green signal pattern indicative of a rearrangement of one copy of the SS18 gene region. These findings were consistent with the diagnosis of synovial sarcoma.

**Figure 2:**
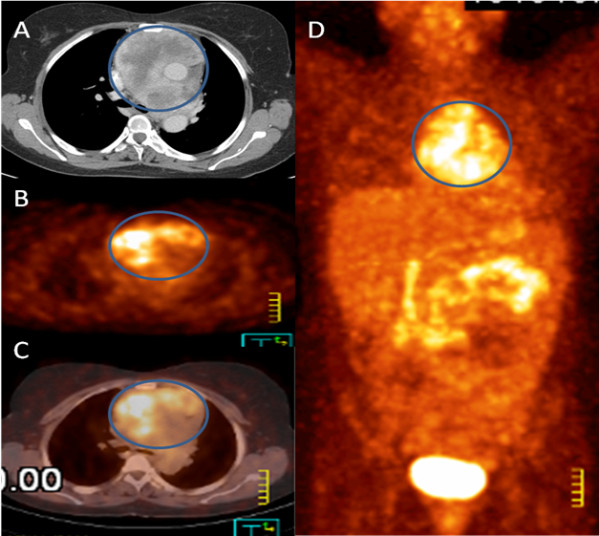
**PET/CT performed for characterization and staging of the tumor.** Axial contrast enhanced CT scan (**A**) showed heterogeneously enhanced anterior mediastinal soft tissue mass (circle) engulfing the major vessels , axial PET (**B**) and PET/CT (**C**) revealed a huge hyper-metabolic mass (circle) in the anterior mediastinum which demonstrated heterogeneous increased FDG metabolic activity with a maximum standardized uptake value (SUVmax) of 6. Maximum intensity projection (MIP) image (**D**) also illustrated this mass (circle) in the coronal dimension. Remainder of the PET/CT exam was negative for any distant hyper-metabolic lesions.

A 35 year old female presented with bilateral lower limb swelling and exertional shortness of breath that had developed over few weeks. She was found to have pericardial effusion that was aspirated at another hospital. The patient was discharged two days later on non-steroidal anti-inflammatory medication after showing subjective improvement. Two weeks later, she had recurrence of her symptoms due to recurrent pericardial effusion with signs of impending tamponade that was managed with a pericardial window. A computed axial tomography (CT) scan of the thorax demonstrated a huge mediastinal mass, for which a CT guided biopsy was carried out. The patient was referred to us later for further evaluation. On presentation she was asymptomatic and her physical examination disclosed mild bilateral pitting edema, but was otherwise noncontributory. A review of her CT scan showed a huge mass in the anterior mediastinum and no evidence of distant metastases. Review of her pathology specimen showed proliferation of spindle cells with oval normochromatic nuclei, scattered mitotic figures and no evidence of necrosis. Prominent thin- walled blood vessels were seen with branching embarking a hemangiopericytoma-like vascular pattern (Figure 1A). No epithelial components could be appreciated. A panel of immunostains was done. The tumor cells were positive for epithelial membrane antigen (EMA), and focally positive for pan-cytokeratin (MNF) (Figure 1B) as well as for BCL-2 (Figure 1C) and FLI-1 (Figure 1D). They were negative for CD99, S100 protein, CD34, CD31, DOG-1, C-kit, CD20 and CD3 immunostains, which essentially excluded other sarcomas, germ cell tumors and lymphomas. The morphological and immunostains supported the diagnosis of synovial sarcoma, monophasic variant, which was confirmed with Fluorescence in situ hybridization (FISH) testing for ss18 gene rearrangement (Figure 1E). A Positron emission tomography/ CT (PET/ CT) scan was carried out and demonstrated the huge hyper-metabolic mass, with a maximum standardized uptake value (SUVmax) of 6, in the anterior mediastinum and ruled out distant metastatic sites (Figure 2).

The mass was unresectable according to the thoracic surgeons’ assessment because it was invading the pericardium and engulfing the major vessels. We elected to proceed with combination chemotherapy in an attempt to downsize her tumor for possible subsequent resection. The option of EBRT was discussed, however; given the huge size of her mediastinal mass, a sufficient and definitive dose of radiation would not be feasible without severe toxicity. She received three cycles of Ifosfamide 2g/m2 every 12 hours D1-D3, Doxorubicin 75 mg/m2, Mesna and Granulocyte-colony stimulating factor (GCSF) starting 24 hours following completion of chemotherapy and continued until neutrophil recovery. She had recurrent neutropenic fever following the first two cycles in addition to severe thrombocytopenia and epistaxis following the second cycle which required 25% dose reduction of both agents in the third cycle. Her chest CT scan following the third cycle showed slight increase in tumor size. The patient refused further therapy and was referred to palliative care. Three months later, she started to suffer from progressive shortness of breath and chest discomfort secondary to mass effect, A CT scan of her chest and abdomen showed progression of the size of the mass without distant metastasis. The patient is still alive with her disease 11 months after diagnosis.

## Discussion

Primary thoracic sarcomas are rare group of neoplasms that may arise in mediastinum, heart, lung, and pleura, and includes a wide variety of possible histologic subtypes. Although angiosarcoma, leiomyosarcoma, sarcomatoid mesothelioma, and rhabdomyosarcoma are the most commonly encountered histologies, other subtypes including synovial sarcoma, fibrosarcoma, malignant fibrous histiocytoma, chondrosarcoma, and primitive neuroectodermal tumors may arise in these locations. Apart from rhabdomyosarcoma which has a bimodal occurrence (children, fifth to seventh decades), almost all of these tumors affect middle aged and elderly patients, and there occurrence in children is exceedingly rare [6].

Morphologically, synovial sarcoma can show several growth patterns and is basically divided into monophasic and biphasic subtypes. The better recognized variant is the biphasic synovial sarcoma which consists of proliferation of bland looking spindle-shaped cells in a collagenous background and hemangiopericytomatous vascular growth pattern, along with evidence of epithelial differentiation ranging from well-formed gland-like structure to aggregates of cudoidal cells. Monophasic tumors on the other hand can show either spindle cells only or occasionally might consist of epithelial component only [7]. A poorly differentiated variant of synovial sarcoma is also recognized [8]. Immunostains has been invaluable in supporting the diagnosis of synovial sarcoma, especially with limited biopsy material as in our case, when the diagnostic features are not well represented, or in the poorly differentiated variant. The expression of epithelial markers in the gland-like component and more importantly in the spindle cell component supports the diagnosis. Among all epithelial markers, EMA is the most commonly positive marker [8]. Pan-cytokeratin, cytokeratin 7, and 19 can also be positive in the epithelial-like component as well as the spindle cell component [9]. In addition; positivity for bcl-2 can be seen in some cases [10]. Recently; TLE1 has been described as a diagnostic marker for synovial sarcoma which is claimed to be more sensitive and specific than others [11].

Although the majority arises in extremities [12], synovial sarcomas can arise in any site including head and neck, abdominal wall, lungs, and genitourinary tract. Nevertheless, the mediastinum as a primary site of occurrence is rare with less than 40 reported cases in literature.

Because the mediastinum is a host for a variety of primary and secondary neoplasms, differentiating synovial sarcomas from variety of other, more commonly encountered diagnoses such as lymphoma, mesothelioma, thymoma, metastatic carcinomas, as well as other sarcoma subtypes is of paramount importance. Careful assessment of biopsy specimen, with adjunctive imunostains is necessary for appropriate diagnosis. The availability of molecular genetic identification (FISH or polymerase chain reaction) of the t(X;18) has improved diagnostic specificity for synovial sarcomas as this translocation is found in over 90% of cases [13]. Testing for this translocation is important to confirm a suspected diagnosis of synovial sarcoma when it arises in a rare location.

Following establishment of diagnosis of a mediastinal or cardiac sarcoma, accurate staging to identify the extent of disease and to rule out distant metastases is an important and essential step for subsequent management.

Apart from rhabdomyosarcoma and primitive neuroectodermal tumors, which require histology-specific chemotherapy regimens as integral part of treatment, management of other histologic subtypes, regardless of age and location, share same general principles. Surgical resection is the cornerstone of therapy of thoracic STS including those arising in the mediastinum if the mass is resectable; in fact, the ability to completely resect the mass is the most important factor associated with long term survival [3].

Although there are no randomized phase III studies to define the most effective modality, patients with unresectable sarcomas are usually treated with systemic chemotherapy and/ or EBRT [14,15]. Because an adequate response to EBRT and chemotherapy may convert an initially unresectable tumor to one that can be resected and potentially cured, the choice of particular type of chemotherapy regimen or of radiotherapy is usually guided by the sarcoma histologic subtype, given the recognized wide variability of response of different STS subtypes to chemotherapy regimens and radiotherapy. Combination chemotherapy in this setting is preferable over single agents because of the higher probability of achieving an adequate response.

For patients presenting with unresectable mediastinal synovial sarcomas, very limited data exists regarding the optimal therapy that can effectively downsize the tumor to resectable state. A review of literature identified only 34 cases of primary synovial sarcoma of the mediastinum, 16 of them presented as unresectable disease (Table 1). The most commonly employed therapeutic modality for patients with unresectable disease is EBRT. There are only four reports where chemotherapy had been the primary therapy, It is worth noting that amongst those patients who presented with unresectable disease, none had subsequent complete surgical resection. Most cases had progressed locally and at distant sites shortly following completion of radiotherapy and chemotherapy and died within two years of diagnosis, with no reported long term survivals.

Unlike patients with unresectable disease, patients whose’ disease can be completely resected had a better chance for long term survival; Gerry Van der Mieren et al. [16], reported a patient who survived more for 14 years with a strategy of repeated surgical resection.

In patients with resectable mediastinal disease, the prognosis seems to be worse than patients with resectable extremity synovial sarcomas, as most of the reported cases of mediastinal synovial sarcomas had local or distant recurrences following complete surgical resection [17,18]. This high rate of local and distant failure suggests that adjuvant multimodality therapy following resection might be warranted.

## Conclusions

Synovial sarcoma arising in the mediastinum is a rare disease entity. Complete surgical resection is the only known factor associated with possible long term survival. When arising as unresectable mediastinal mass, the prognosis is extremely poor. Reports of effective downsizing strategies and studies addressing the best integration of chemotherapy and radiotherapy in patients with unresectable mediastinal disease are warranted. Our case can be of benefit to medical oncologists and thoracic surgeons who might be faced with this unique and exceedingly rare clinical scenario.

## Consent

Written informed consent was obtained from the patient for publication of this case report and accompanying images. A copy of the written consent is available for review by the Editor-in-Chief of this journal.

## Abbreviations

CT: Computed axial tomography; EBRT: External beam radiotherapy; FISH: Fluorescence in situ hybridization; PET: Positron emission tomography; SUVmax: maximum standardized uptake value; GCSF: Granulocyte-colony stimulating factor; STS: Soft tissue sarcomas.

## Competing interests

The authors declare that they have no competing interests.

## Authors’ contributions

SS wrote the paper, reviewed the literature, constructed the table, cared for the patients, and approved the final version of the manuscript. AA interpreted the imaging studies, assisted in writing the paper, and provided the radiology image. AD performed the FISH study and provided an image and a descriptive legend for the FISH study. MA reviewed the pathology, performed the immunostains, assisted in writing the paper, and provided the pathology image and a descriptive legend. All authors read and approved the final manuscript.
